# Spectrum of CT appearance and CT severity index of COVID-19 pulmonary infection in correlation with age, sex, and PCR test: an Iraqi experience

**DOI:** 10.1186/s43055-021-00422-3

**Published:** 2021-01-29

**Authors:** Ammar Mosa Al-Mosawe, Hiba mohammed Abdulwahid, Noor Abbas Hummadi Fayadh

**Affiliations:** 1 College of Medicine, Al-Nahrain University, Home no. 12/1, Street no. 27/, Al-Masbah locality 929, Baghdad, Iraq; 2 College of Medicine, University of Baghdad, Baghdad, Iraq

**Keywords:** COVID-19, Chest CT, CT severity score index, PCR

## Abstract

**Background:**

Since June 2020, an explosion in number of new COVID-19 patients has been reported in Iraq with a steady increment in new daily reported cases over the next 3 months. The limited number of PCR kits in the country and the increment in the number of new COVID-19 cases makes the role of CT scan examinations rising and becoming essential in aiding the health institutions in diagnosing and isolating infected patients and those in close contacts. This study will review the spectrum of CT pulmonary changes due to COVID-19 infection and estimate the CT severity score index and its relation to age, sex, and PCR test results**.**

**Results:**

The ground glass opacities were the most common encountered pattern of pulmonary changes and were seen in (79%). There was strong positive correlation between higher CT severity score and male gender (*p* value = 0.0002, *R*^2^ = 0.9). Also, there was significant correlation of CT severity score and increasing age (*p* value less than 0.00018). Significant correlation was seen between CT scan percentage of lung involvement and positive PCR test results (*p* value = 0.001917), as the CT severity index is increasing, the PCR test is more likely to be positive.

**Conclusions:**

Chest CT is an important and fast imaging tool for the diagnosis of COVID-19-infected patients especially in developing countries. In addition, chest CT can predict the disease severity by showing the percentage of lung involvement and hence give an idea about the prognosis of the disease. Higher CT severity score is significantly correlated with male gender, older age group patients and likely positive PCR test.

## Background

COVID-19 is a pandemic viral infectious disease caused by a strain of corona virus (SARS-CoV-2). First recorded cases were in Wuhan, China, in the late 2019 then spread globally all over the world. The disease was announced as pandemic by the World Health Organization in 11 March2020 [1].

Regarding the diagnosis of COVID-19 infection, PCR test based on nasal or throat swab sampling is now considered the most effective and appropriate technique for quick COVID-19 screening. It is reported that the approximate RT-PCR test sensitivity for COVID-19 infection is about 50–62% which is a satisfactory result, but still has some substantial number of missed diagnosis [2]. The study of He et al. showed that RT-PCR test for the disease resulted in a sensitivity of 79%, which is higher than those reported previously [2]. The accuracy of RT-PCR results is affected by number of issues, and these include the respiratory tract viral load, samples source, the procedures, and timing of samples acquisition, as well as the intrinsic features and quality of the testing kits [3]. Consequently, the RT-PCR test alone would be debatable to be an independent and solely tool for screening of COVID-19 suspected individuals and hence complementary tools like clinical picture, CT scan examination, and blood tests need indeed to participate in the screening and diagnosis of COVID-19 infection in addition to the PCR test examination [2].

The initial concern about using CT scan in patients with COVID-19 infection was to assess the spectrum of imaging findings and recognizing which pattern being typical or atypical for the disease, and hence many classifications had been emerging to standardize reporting into typical, indeterminate, and atypical CT patterns for COVID-19 [4].

The CT severity score index is a scoring system used to assess the lung changes and involvement by COVID-19 based on approximate estimation of pulmonary involved areas. Each of the five lung lobes has been visually scored and given a score from 1 to 5:

- 1 representing less than 5% lobar involvement.
- 2: 5–25% lobar involvement.
- 3: 26–50% lobar involvement.
- 4: 51–75% lobar involvement.
- 5: > 75% lobar involvement.

Then, the final score will be the summation of individual lobar scores and will be out of 25 (total score); the total lung involvement is then obtained by multiplying the total score times 4 [5].

Since June 2020, an explosion in the number of new COVID-19 positive patients has been reported in Iraq with steady increase in new daily reported cases over the next 3 months [6].

The limited number of PCR kits in Iraq and the increment in the number of new COVID-19 cases makes the role of CT scan examinations rising and becoming essential in aiding the governmental health care providers and health institutions in diagnosing and isolating the infected patients and makes it easier in assisting tracking those in close contacts and hence isolating people with possible disease and community burden. This study will show the spectrum of CT findings of COVID-19-infected Iraqi patients and calculate the severity score for lung involvement of each and its correlation with age, sex, and PCR test.

### Aim of the study

To study the spectrum of CT pulmonary changes due to COVID-19 infection and estimating the CT severity score index and its relation to age, sex, and PCR test results.

## Methods

### Study design

A prospective cross-sectional analytic study included 172 patients, and they were 100 males and 72 females with the age range of 20–85 years, conducted in a single private center in Baghdad/Iraq during the period from 5 August to 9 September 2020.

### Inclusion criteria

1. Symptomatic patients (having one or more of the following; fever, sore throat, cough, shortness of breath) with positive pulmonary CT findings.
2. Patients had history of recent contact with confirmed COVID-19-infected patients and with positive pulmonary CT findings.
3. Available PCR results for all patients (mentioned in 1 and 2) whether the result is positive or negative.

### Exclusion criteria

- Suspected patients with COVID-19 infection but with normal chest CT scan.
- Patient whom their PCR results were unobtainable.
- Patients with PCR results available but time interval between PCR and chest CT scan more than 1 week.

### Patients

After eligibility to the inclusion and exclusion criteria, 172 patients with positive CT pulmonary changes and with available PCR results were included in the study.

### Imaging technique

All patients underwent native chest CT examination in Baghdad at Vision Private Center using Philips GEMINI TF 64 slice CT with the following examination parameters:

Axial sections with 3 mm slice thickness and FOV of 400 mm were obtained. Tube current of 20–30 mA, tube voltage of 120–140 kV, matrix of 512, pitch 1.078, and rotation time of 0.75 s. Sagittal and coronal reformatted images were subsequently obtained. No contrast material was used.

### Image analysis

All CT scan images were analyzed and reviewed by a specialist radiologist. The spectrum of CT findings were: ground glass patches, consolidations, vascular dilatation, crazy paving, subpleural bands, and architectural distortion.

The severity score was calculated based on lung involvement percentage for each patient by scoring the percentage of each lobe involvement individually and given a score from 1 to 5 where

Score 1 representing < 5% involvement

Score 2: 5–25% involvement

Score 3:26–50% involvement

Score 4: 51–75% involvement

Score 5: > 75% involvement

Then, the final score will be the sum of individual lobar scores and will be out of 25 (total score); the total lung involvement is then obtained by multiplying the total score times 4.

### Statistical analysis

The collected data were tabulated and analyzed using Microsoft Excel 2013.

The categorical data were presented as frequency and percentage tables.

Regression analysis was used to calculate the *p* value; statistically significant results were accepted at *p* value < 0.05.

Pearson correlation test was used to evaluate the correlation of total CT-severity score and patient age.

Categorical correlation analysis was used to assess the relationship of CT severity score and the patient gender.

## Results

### Demographic characteristics of patients

This study comprised 172 patients with positive CT findings suspicious for COVID-19 pulmonary infection. They were 100 males (58.2 %) and 72 females (41.8%) with ages range 20–85 years (mean age of 50.3 years). The age group was categorized into 3 groups: Those with less than 30 years of age; between 30 and 60 years and those above 60 years (Table 1).

**Table 1:** Age and sex distribution of patients with COVID-19 pulmonary infection

**Sex**	**No.**	**Percentage %**
Male	100	58.2
Female	72	41.8
Total	172	100
**Age group**	**No.**	**Percentage %**
Below 30 years	18	10.5%
30–60 years	104	60.5%
Above 60 years	50	29%

### The spectrum of CT findings and CT severity index

The ground glass opacities were the most common encountered pattern of pulmonary changes and were seen in (79%), associated vascular enlargement was seen in 121 patients (70%), pulmonary consolidations in 58 patients (33 %). Other findings like subpleural bands were seen in 65 patients (37 %), pulmonary architectural distortion in 59 patients (34%), while crazy paving appearance was evident in 28 patients (16%) (Table 2).

**Table 2:** Spectrum of CT findings in patients with COVID-19 pulmonary infection

CT findings	No.	Percentage %
Ground glass opacities	136	79
Vascular enlargement	121	70
Consolidation	58	33
Subpleural band	65	37
Architectural distortion	59	34
Grazy paving	28	16

### Correlation of CT severity index with age and sex

This study showed that there was strong positive correlation of increment in CT severity score and male sex (*p* value = 0.0002 and *R*^2^ = 0.9).

Two out of 2 patients with score 5 and 8 out of 9 patients with score 4 CT lung involvement were male patients. The results are summarized in Table 3.

**Table 3:** Correlation between CT severity index of patients with COVID-19 pulmonary infection and sex

CT severity index		Male	Female	
	No.	No.	No.	
**Score 1 (< 5%)**	6	2	4	***p*** **value = 0.0002** ***R***^**2**^ **= 0.9**
**Score 2 (5–25%)**	91	53	38	
**Score 3 (26–50%)**	64	35	29	
**Score 4 (51–75%)**	9	8	1	
**Score 5 (> 75%)**	2	2	0	

Also, there was significant correlation of CT severity score and increasing age (*p* value = 0.00018); 8 patients out of total 11 who had total lung involvement of more than 50% were above 60 years of age (Table 4).

**Table 4:** Correlation between CT severity index and age groups of patients of COVID-19 pulmonary infection

CT severity index	Below 30 years (*n* = 18)	30–60 years (*n* = 104)	Above 60 years (*n* = 50)	*p* value
Score 1 (*n* = 6)	5	1	0	0.00018
Score 2 (*n* = 91)	10	60	21	
Score 3 (*n* = 64)	3	40	21	
Score 4 (*n* = 9)	0	3	6	
Score 5 (*n* = 2)	0	0	2	

### Correlation between CT severity index and PCR tests

**Fig. 1:**
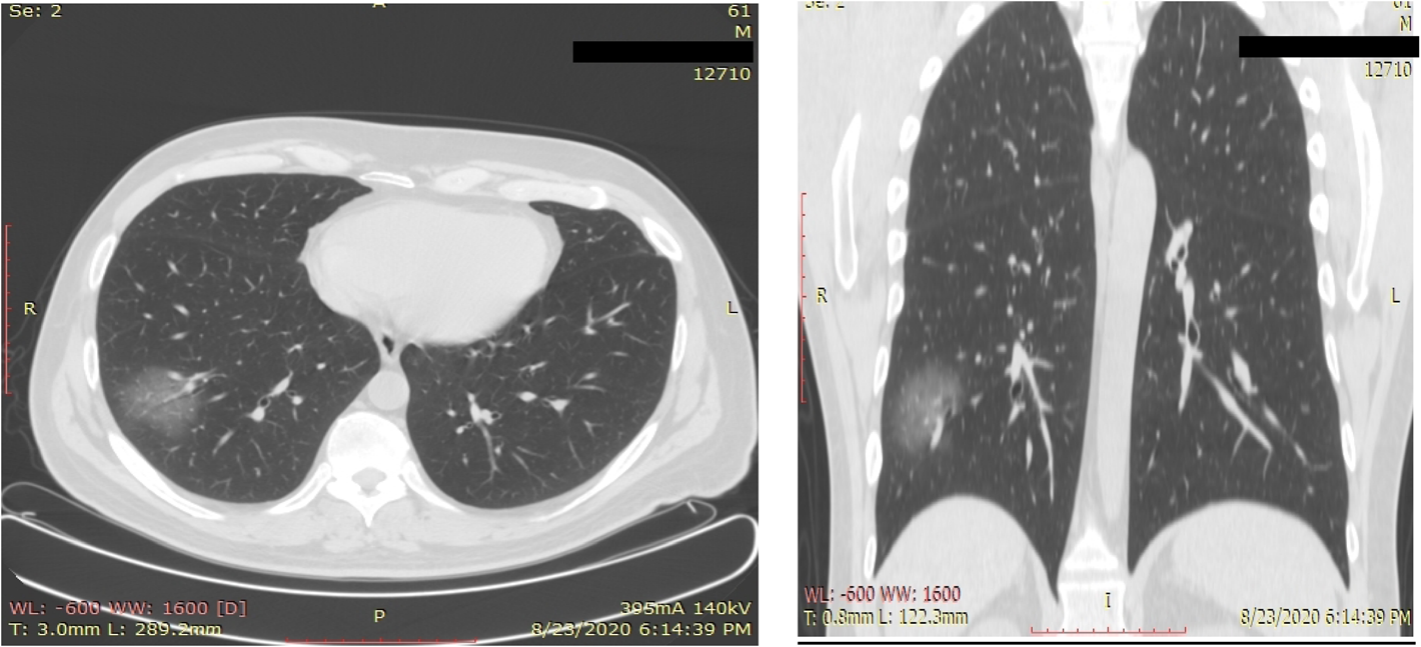
Thirty-two-year-old female patient presented with sore throat and mild fever. Her PCR test was negative, and the CT revealed unifocal ground glass opacity peripherally located in the right lower lobe. Lung involvement was less than 5%, i.e., CT severity score is 1

**Fig. 2:**
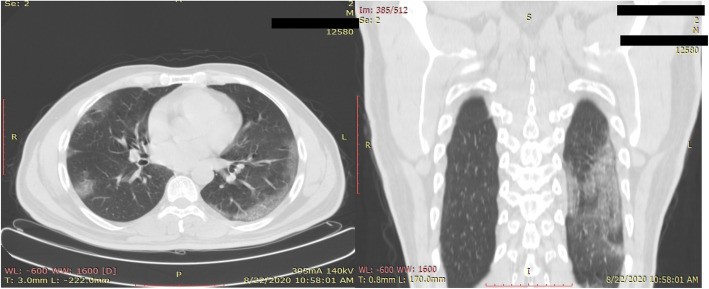
A 36-year-old male patient with positive PCR test. Chest CT shows bilateral ground glass opacities in a peripheral/subpleural distribution with lung involvement of 5–25%, i.e., CT severity score of 2

Significant correlation was seen between CT scan percentage of lung involvement and positive PCR test results (*p* value = 0.001917), as the CT severity index is increasing, the PCR test is more likely to be positive. Six patients show less than 5% of CT lung involvement (i.e., score 1); 3 of them only (50%) show positive PCR test (Fig. 1); while 91 patients were recorded with 5–25% lung changes (i.e., score 2) and 58 of them (about 63%)showed PCR-positive test (Fig. 2).

Fifty-two out of total 64 patients with 26–50% involvement (score 3) tested positive for COVID-19 on PCR (i.e., 82%).

**Fig. 3:**
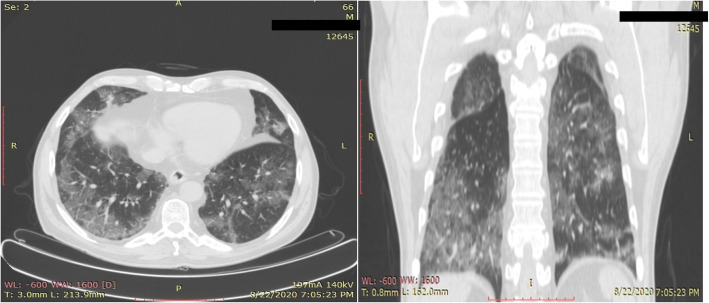
Chest CT of a 66-year-old male patient showing bilateral ground glass pulmonary changes predominantly in peripheral distribution. The lung involvement was 50–75% and the CT severity index score was 4. The PCR test for COVID-19 was positive for this patient

**Fig. 4:**
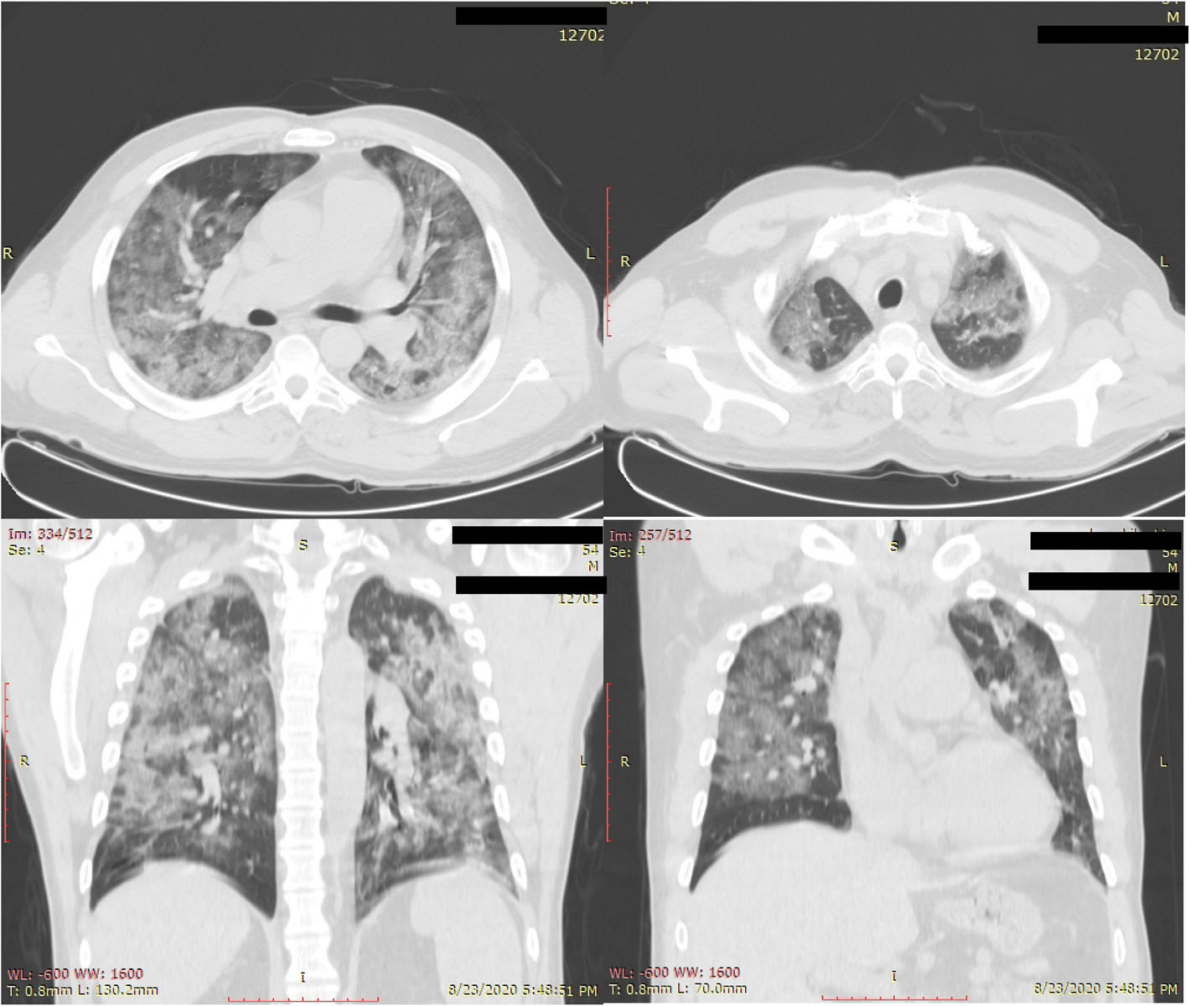
Sixty-four-year-old male presented with cough. His PCR test was positive. CT chest is showing diffuse ground glass opacities with consolidative changes. There is associated vascular enlargement within the GG areas. Lung involvement is about 80%, i.e., CT severity score is 5

Fifty-one to 75% lung involvement (score 4; Fig. 3) was seen in 9 patients; of them, 8 were tested PCR-positive, while both of the 2 patients who showed more than 75% total lung involvement (i.e., score 5; Fig. 4) were PCR-positive for COVID-19.

This gives a total of 123 patients who showed positive laboratory PCR test for COVID-19 and that 49 patients were negative for the test (Table 5).

**Table 5:** Correlation between CT severity index and PCR results

CT severity index	No.	+VE PCR	−ve PCR	***p*** value
		No.	No.	
**Score 1 (< 5%)**	6	3	3	**0.001917**
**Score 2 (5–25%)**	91	58	33	
**Score 3 (26–50%)**	64	52	12	
**Score 4 (51–75%)**	9	8	1	
**Score 5 (> 75%)**	2	2	0	

## Discussion

The current study showed that about 58.2% of patients were male and 41.8% were female and that the more severe CT pulmonary changes (i.e., higher CT severity score) was significantly associated with male gender. This was in agreement with many other studies that showed that COVID-19 infection affect males more than females and the former tend to have a more severe form of the disease and this might be attributed to the biological differences between men and women [7, 8]. One of these studies was that done by Borghesi et al. who studied 783 Italian patients and found that higher CXR score was significantly higher in males than in females [8].

The more severe CT lung changes tends to be significantly higher in older age group (above 60 years of age) with significant correlation (*p* value = 0.00018); this may be attributed to the coexisting morbidities in those patients and other factors related to aging. This was in agreement to the results reported by Liu et al [9]. It is also valuable to note that COVID-19-infected patients who need hospitalization, RCU admission, and with higher mortality rate were more likely of male gender and those with higher age group populations [8, 10].

The current study revealed that the ground glass opacities were the most common encountered pattern of pulmonary changes and were seen in 136 patients (79%) with associated vascular dilatation seen in 70% of cases and seen within the areas of ground glass patches. This was similar to many other studies, Parry et al. [11], Omar et al. in Egypt [7], and Adnan et al. [12], where ground glass pattern was the most common CT pattern in their studies. Intralesional vascular enlargement was encountered in 70% in the study sample of Parry et al. [11].

Consolidation was encountered in 33% in this study; this is in comparison to the findings reported by Omer et al. [7] and Adnan et al. [12] who recorded consolidation in 23% and 9% in their studies respectively; this is may be attributed to the timing at which CT examination is performed as consolidation with or without ground glass changes will be seen in the 2nd and 3rd weeks of infection course.

As with many previous literatures, pleural effusions or cavitary lesions were not encountered in this study [7, 11, 12].

This study revealed that among the 172 studied patients with positive CT findings, only 123 of them (71.5%) had positive PCR test for COVID-19. This was in agreement to the studies of He et al. who found that 27 patients had positive PCR results for COVID-19 out of total 34 patients (i.e., about 79%) in their study [2] and Yang et al. [13].

The study of Fang et al. [14] support the use of CT for screening of COVID-19 in patients who exhibit epidemiologic and clinical features compatible with COVID-19 infection, especially when the RT-PCR tests results are negative [14].

Ai et al. who studied the correlation of chest CT and PCR test for COVID-19 in 1014 patients in china concluded that CT chest might be considered as a primary imaging technique for the detection of COVID-19 in epidemic areas [15].

There was a significant association between CT severity score index and positive PCR test (*p* value = 0.0019), as the CT severity index is increasing, the PCR test is more likely to be positive. This will predict the positivity of PCR with increasing severity of pulmonary involvement by CT (i.e., higher CT score index). To the best of our knowledge, no previous literatures had studied such correlation between the increment in CT severity score index and PCR test results.

## Conclusions

Chest CT is an important and fast imaging tool for the diagnostic work up of COVID-19-infected patients especially in developing countries like Iraq, where the PCR testing kits are not widely available and not covering the marked increment in number of newly infected cases; furthermore, the results of PCR tests may be delayed for several days.

Chest CT scan can predict the severity of the disease by showing the percentage of lung involvement and hence give an idea about the prognosis of the disease so that an appropriate and optimal management would be overtaken earlier, thus decreasing patient hospitalization and mortality rate.

Higher CT severity score is positively correlated with male gender and older age group patients.

The PCR test is more likely to be positive with increasing CT severity score index.

## Data Availability

All data used in this study are available with the corresponding author on reasonable request.

## Abbreviations

CT: Computerized tomography
FOV: Field of view
PCR: Polymerase chain reaction
RT-PCR: Reverse transcriptase polymerase chain reaction
